# Age-specific prevalence of HPV genotypes in cervical cytology samples with equivocal or low-grade lesions

**DOI:** 10.1038/sj.bjc.6605165

**Published:** 2009-07-21

**Authors:** S Brismar-Wendel, M Froberg, A Hjerpe, S Andersson, B Johansson

**Affiliations:** 1 Department of Clinical Science, Intervention and Technology, Division of Obstetrics and Gynaecology, Karolinska University Hospital Huddinge, Karolinska Institutet, 141 86 Stockholm, Sweden; 2 Centre for Clinical Research (CKF), Uppsala University, Centrallasarettet, 721 89 Västerås, Sweden; 3 Department of Laboratory Medicine, Division of Pathology, Karolinska University Hospital Huddinge, Karolinska Institutet, 141 86 Stockholm, Sweden; 4 Department of Laboratory Medicine, Division of Clinical Virology, Karolinska University Hospital Huddinge, Karolinska Institutet, 141 86 Stockholm, Sweden

**Keywords:** HPV, genotyping, liquid-based cytology (LBC), ASCUS, LSIL, age

## Abstract

**Background:**

To define the spectrum of human papillomavirus (HPV) types and establish an age limit for triage HPV testing in atypical squamous cells of undetermined significance (ASCUS) and low-grade squamous intraepithelial lesion (LSIL).

**Materials and methods:**

343 liquid-based cytological samples from the population-based screening programme with minor abnormalities were subjected to HPV genotyping (Linear Array, Roche, Basel, Switzerland).

**Results:**

High-risk human papillomavirus (HR-HPV) was found in 71% of LSIL and 49% of ASCUS cases (*P*<0.001). High-risk human papillomavirus prevalence was age-dependent in LSIL (*P*=0.01), with decreasing prevalence until the age of 50 years, followed by a slight increase. Human papillomavirus type 16 was the most common HR-HPV, found in 23% of HPV-positive women. Human papillomavirus type 18 was the sixth most common, found in 9.9% (*P*<0.001). An age-dependent quadratic trend was observed for multiple infections (*P*=0.01) with a trough at about 42 years. The most common HR-HPV types to show a coinfection with HPV16 (clade 9) were HPV39 (28%), 45 (38%), and 59 (46%), belonging to HPV18 clade 7. The frequency of low-risk (LR) *vs* probable HR and HR-HPV also followed an age-dependent quadratic trend.

**Conclusions:**

After the age of 25 years, HR-HPV prevalence is similar in LSIL and ASCUS cases, motivating a low age limit for triage HPV testing. Multiple infections and LR/HR-HPV dominance are age-dependent. Genotyping in longitudinal design is needed to elucidate the importance of multiple infections in cancer progression and in cross-protection from vaccination.

Persistent infection with high-risk human papillomavirus (HR-HPV) types has a critical aetiological function in the development of cervical intraepithelial neoplasia (CIN) and cervical cancer (Walboomers *et al*, 1999). Progression from low-grade to high-grade dysplasia and invasive disease is rare in the absence of HPV (Khan *et al*, 2005). Human papillomavirus types16 and 18 are incriminated as the causative agents in 65–77% of cervical cancers (Smith *et al*, 2007). The effect of multiple HPV infections on CIN and cancer development is still unclear, but several publications argue that certain multiple infections may confer a risk similar to the risk of a single HPV16 infection (Becker *et al*, 1994; Trottier *et al*, 2006; Wheeler *et al*, 2006). With the implementation of vaccination programmes, interest is also growing in cross-protection against HPV genotypes belonging to the same clade or phylogenetic species (Ault, 2007; Castellsague *et al*, 2008; Poolman *et al*, 2008; Sargent *et al*, 2008). Human papillomavirus type 16 belongs to clade 9, as do HPV31, 33, 35, 52, and 58, whereas HPV18 belongs to clade 7, along with HPV39, 45, 59, and 68 (de Villiers *et al*, 2004).

Awareness of the causal relationship between HPV infection and cervical cancer has made the detection of the virus an attractive approach to identify women at risk of developing cervical cancer (Cuzick *et al*, 2008). This approach, which is particularly important in cases of minor cytological abnormalities, is internationally accepted for use in cases of atypical squamous cells of undetermined significance (ASCUS). However, it has not been generally recommended in cases of low-grade squamous intraepithelial lesions (LSILs) because of the high prevalence of oncogenic HPV in this group (Sherman *et al*, 2002; Schiffman and Solomon, 2003; Arbyn *et al*, 2006; Wright *et al*, 2007). Women with minor cytological abnormalities account for a large proportion of those referred for further investigation, a process resulting in psychological stress and considerable health-care costs (Freeman-Wang *et al*, 2001; Idestrom *et al*, 2003).

In Sweden, about 3–5% of all smears show some kind of abnormality, almost 80% of which are minor cytological changes (Sparen, 2008). Current Swedish guidelines for management of minor abnormalities do not include HPV testing; rather, a second Pap smear or colposcopy is required (Ahlberg-Ranje *et al*, 2008). The question whether HPV testing may improve the Swedish screening programme is under discussion, especially in the light of the potential to identify precancerous lesions among ASCUS and LSIL and to distinguish these from reactive or degenerative lesions. According to our previous study, complementing liquid-based cytology (LBC) with an HPV test in cases of ASCUS and LSIL may lead to a 40% decrease in the number of these women referred for further investigation (Froberg *et al*, 2008). Human papillomavirus prevalence is high among younger women with LSIL, which is why an age limit for triage HPV testing is being sought. We wished to compare the age-specific prevalence of HR-HPV in these cytological categories to show that difference in prevalence is not an argument against triage testing.

The purpose of our study was to identify the HPV types present among women with minor cytological abnormalities using the PCR-based Linear Array HPV Genotyping Test (Roche Diagnostics, Basel, Switzerland) and to describe the prevalence of multiple infections in relation to age before the implementation of vaccination programmes.

## Materials and methods

### Patients

From September 2005 through to September 2006, 4204 LBC samples from a population-based screening programme were obtained from seven maternity health centres in Southern Stockholm as previously reported (Froberg *et al*, 2008). Later in 2006, an additional 2024 samples were collected. Minor cytological abnormalities were found in 343 (5.5%) of these 6228 LBC samples, comprising the cytological classifications of ASCUS and LSIL based on the Bethesda nomenclature system (Solomon *et al*, 2002). According to Swedish recommendations, koilocytosis without signs of dysplasia was reported as non-pathologic. Therefore, the LSIL group only contains samples corresponding to cytological cervical intraepithelial neoplasia grade 1 (CIN1). Approval from the ethics committee and written informed consent from all women were obtained before inclusion.

### HPV DNA extraction and detection with linear array

A 2 ml PreservCyt suspension from the remaining LBC sample after cytological evaluation was centrifuged and the cell pellet was lysed according to instructions in the Total Nucleic Acid Isolation kit. DNA was extracted using the MagNA Pure LC robot and analysed with the Linear Array HPV Genotyping Test (LA), according to the manufacturer's instructions (all procedures by Roche). The method includes a PCR amplification of extracted HPV DNA using a pool of biotinylated primers that hybridise in the L1 region of the HPV genome, chemical denaturation of HPV DNA amplicons to single-stranded DNA, followed by hybridisation with matching type-specific DNA probes immobilised on nylon strips, and detection by colorimetric determination. The result is a pattern of blue lines on a nylon strip, which is visually read by comparing the pattern with a reference guide. The LA test covers 37 HPV types including 12 HR-HPV types (16, 18, 31, 33, 35, 39, 45, 51, 52, 56, 58, 59), 6 probable high-risk (pHR-) HPV types (26, 53, 66, 68, 73, 82), and 19 low-risk or undetermined-risk (LR-) HPV types (6, 11, 40, 42, 54, 55, 61, 62, 64, 67, 69, 70, 71, 72, 81, 83, 84, IS39, CP6108) (Munoz *et al*, 2003).

### Statistical analysis

Data were analysed with the software STATISTICA 6.1 (Statsoft Inc, Tulsa, OK, USA). Pearson's *χ*^2^ and Yates corrected *χ*^2^ (for *n*<5) tests were used to compare proportions, and Student's *t*-test was used to compare continuous variables between two groups. After plotting proportions of HR-HPV- and HPV-infected women and multiple infections, respectively, in each age group, a linear or polynomial fit was visually assessed to be the best model for logistic regression. We tested whether the proportion in each age group decreased linearly with age (log(p/1−p)=b_0_−b_1_x) and whether this was significantly different from null. Then we added a second degree (log(p/1−p)=b_0_−b_1_x+b_2_x^2^) to test whether the increase in women over a certain age was significantly different from null. We controlled for covariation between age and age^2^ using centred age and centred age^2^ in the model. The larger model with a second degree provided a better explanation as tested with Nagelkerke *R*^2^. Therefore, we concluded that the quadratic function, log(p/1−p)=b_0_−b_1_x+b_2_x^2^, could best describe the relationship between prevalence of HR-HPV in LSIL cases and age, multiple infections and age, and HR/LR-HPV dominance and age. Logistic regressions were also carried out using age group and cytological diagnosis as categorical predictors, as well as HPV prevalence as the dependent variable. The null hypothesis of no difference was rejected at a significance level of *P*<0.05.

## Results

### Cytology results and age distribution

There were 223 cases of LSIL and 120 cases of ASCUS, including three cases of atypical glandular cells of undetermined significance (AGUS). Age was illegible on one LSIL sample and on one ASCUS sample; therefore age was recorded in 341 of 343 women. The mean age was 33.6 years (median 32, range 22–60). Women were grouped according to age at 5-year intervals from age 20–49 years, whereas the oldest women, aged 50–60 years, were placed into a single comparably sized group. Table 1 shows age distribution. The mean age of women with ASCUS was 35.7 years and of those with LSIL was 32.4 years (*P*=0.002). In all, 73% of the women were younger than 40 years. Women with ASCUS were older than women with LSIL; 38% of women with ASCUS were 40 years or older, compared with 21% of women with LSIL (*P*<0.001, *χ*^2^).

### HPV prevalence

The *β*-globin gene from all 343 samples was successfully amplified as shown by the presence of both low- and high-concentration bands on the array. A total of 282 (82%) women tested positive for one or more of the 37 included HPV types. Human papillomavirus prevalence ranged from 92% in the 20–29-year age group to 62% in women aged 40–45 years. Prevalence was 67% in women over 50 years. Age-specific prevalence for all HPV types and HR-HPV types (12 or 13 types) was higher in younger age groups and there was a linear decline with age (*P*<0.001, logistic regression, Table 1).

**Figure 1:**
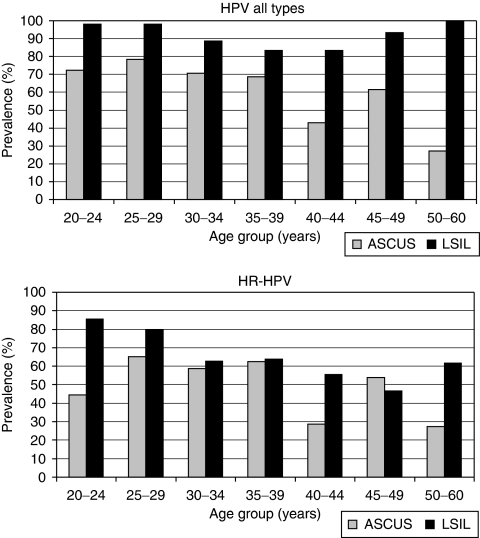
Age-specific prevalence of human papillomavirus (HPV) (all types) and HR-HPV infection in women with ASCUS and LSIL. Human papillomavirus all types *P*(age group × cytological diagnosis)=0.10; LSIL *P*(age)=0.20, *P*(age group)=0.03, *P*(age^2^)=0.003; ASCUS *P*(age)=0.001, *P*(age group)=0.04, *P*(age^2^)=0.34; HR-HPV *P*(age group × cytological diagnosis)=0.15; LSIL *P*(age)=0.001, *P*(age group)=0.01, *P*(age^2^)=0.19; ASCUS *P*(age)=0.04, *P*(age group)=0.11, *P*(age^2^)=0.16; all *P*-values calculated by logistic regression.

Human papillomavirus was more common in women with LSIL (207 out of 223, 93%) than in women with ASCUS (75 out of 120, 63%, *P*<0.001, *χ*^2^). Age-specific prevalence of all HPV types in women with ASCUS compared with women with LSIL is shown in Figure 1. Human papillomavirus prevalence was significantly dependent on age group (*P*=0.03), but did not reach a statistically significant difference between ASCUS and LSIL within each age group using logistic regression (*P*=0.098). However, there is an apparent tendency for increasing discrepancy with lower and especially higher age. *χ*^2^-tests to compare proportions gave significant differences in all age groups, except in the 30–39 year age group.

High-risk or pHR-HPV (18 types) was found in 68 (57%) ASCUS cases, compared with 181 (81%) LSIL cases (*P*<0.001, *χ*^2^). High-risk human papillomavirus (12 types) was found in 59 (49%) cases among women with ASCUS, compared with 158 (71%) in women with LSIL (*P*<0.001, *χ*^2^). Considering 13 types (HR-HPV and pHR-HPV 68), these were found in 60 (50%) ASCUS cases and in 159 (71%) LSIL cases (*P*<0.001, *χ*^2^). Figure 1 shows that HR-HPV prevalence was age dependent among women with LSIL (*P*=0.001, logistic regression), with a decreasing prevalence until the age of 50 years. A small increase was found in older women, but this was not statistically significant (*P*=0.19 for age^2^). A linear correlation between HR-HPV prevalence and age (but not age group) was also found in ASCUS cases (Figure 1). Age-specific HR-HPV prevalence did not reach a statistically significant difference between ASCUS and LSIL using logistic regression (*P*=0.15). *χ*^2^-tests revealed a significant difference in HR-HPV prevalence between ASCUS and LSIL in the youngest age group (Table 1).

### HPV genotype distribution

**Figure 2:**
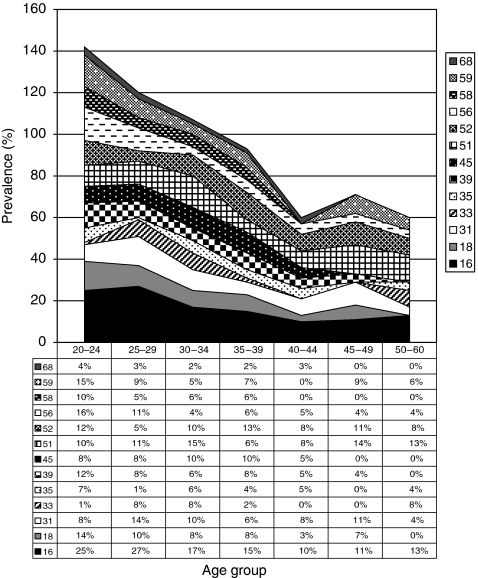
Age-specific distribution of the HR-HPV genotypes including pHR-HPV68 in all women. The total exceeds 100%, as many women carry more than one HPV genotype.

**Figure 3:**
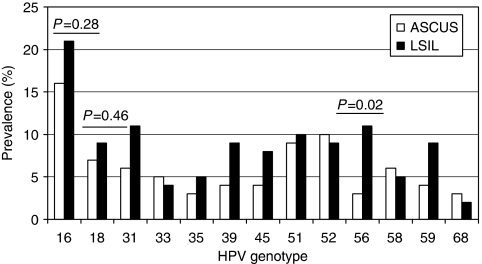
Prevalence of the most common HR-HPV (including pHR-HPV68) genotypes in all LSIL and ASCUS cases.

**Figure 4:**
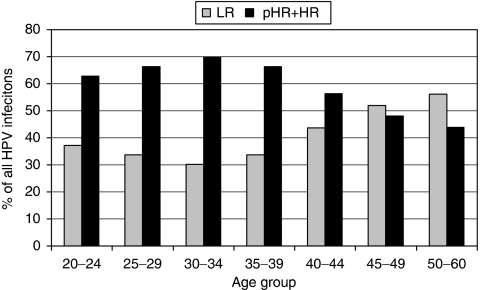
Age-dependent shift in LR *vs* pHR+HR frequency (% of all HPV infections). LR and pHR+HR *P*(age^2^)<0.001 (logistic regression). LR=low-risk HPV, pHR=probable high-risk HPV, and HR=high-risk HPV.

Figure 2 shows the prevalence of HR-HPV genotypes (including pHR-HPV68) in each age group in all women. Human papillomavirus type 16 was the most common HR-HPV and was found in 65 (23%) of the HPV-positive women, ranging from 30% (20 out of 67) in women aged 25–29 years to 14% (3 out of 22) in women aged 45–49 years (*P*=0.22, *χ*^2^). Remarkably, HPV18 was only found in 28 (9.9%) of all HPV-positive women (*P*<0.001 compared with HPV16, *χ*^2^). Human papillomavirus types 51, 52, 56, and 31 were also more common than HPV18. Human papillomavirus type 51 was equally or more common than HPV16 among women over 45 years of age. Among women with ASCUS, the most prevalent HR-HPV genotype was HPV16 (16%), followed by HPV51 and 52 (10%). Among women with LSIL, HPV16 was detected in 21%, followed by HPV56 in 11% of women (Figure 3). Table 2 outlines women infected with HPV16, 18, clade 9 HPV types, and clade 7 HPV types to elucidate the preventive potential of current HPV vaccines against low-grade cytological abnormalities. As shown in Figure 4, we found a shift in predominant genotypes with age. The proportion of LR genotypes increased with age, following a quadratic function (*P*<0.001, logistic regression).

### Multiple infections

**Figure 5:**
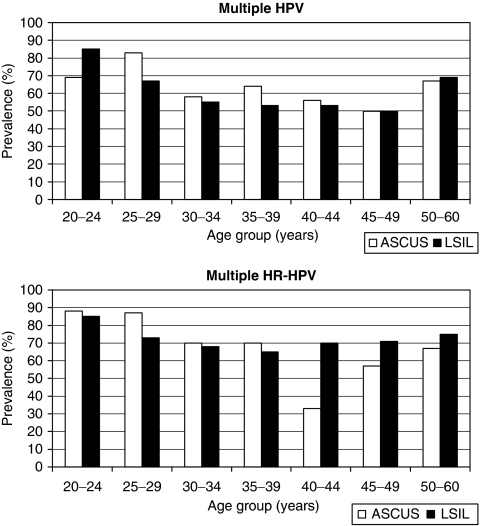
Age-specific prevalence of multiple infections. Multiple HPV *P*(age group)=0.08, *P*(cytological diagnosis)=0.63, *P*(age group × cytological diagnosis)=0.73, LSIL *P*(age^2^)=0.001, turning point 41.6 years, ASCUS *P*(age^2^)=0.76. Multiple HR-HPV *P*(age group)=0.04, *P*(cytological diagnosis)=0.71, *P*(age group × cytological diagnosis)=0.24, LSIL *P*(age^2^)=0.04, turning point 38.6 years, ASCUS *P*(age group)=0.27, *P*(age^2^)=0.34. All *P*-values calculated by logistic regression.

Multiple infections were common in our material (Figure 5). On average, we found 2.5 genotypes per HPV-positive sample. In one LSIL sample, nine different genotypes were found. In all, 54% (185 out of 343) of all women carried more than one HPV type. Women with LSIL had multiple types (61%, 136 out of 223) more frequently than did women with ASCUS (41%, 49 out of 120, *P*<0.001, *χ*^2^). Taking only HPV-positive samples into account, multiple infections were equally common in women with ASCUS and LSIL (65% or 49 out of 75, and 66% or 136 out of 207, respectively). The age-specific prevalence of multiple infections followed a quadratic function in LSIL cases (*P*=0.001, logistic regression, Figure 5). This was also found in multiple HR-HPV infections among the HR-HPV-positive LSIL cases (*P*=0.04, logistic regression). Among HR-HPV-positive samples, 39% (85 out of 217) were infected by more than one HR-HPV type and similar results were found for LSIL and ASCUS samples (39.9 and 37.3%, respectively, *P*=0.73, *χ*^2^).

**Figure 6:**
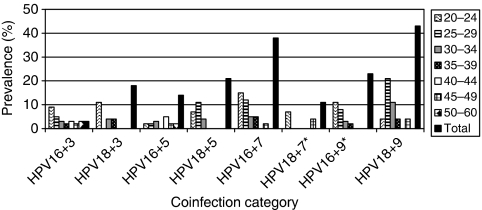
Coinfection with HPV16 or HPV18 and clade 3, 5, 7, and 9. Clade 3: HPV61, 72, 81, 83, 84; clade 5: HPV26, 51, 69, 82; clade 7: HPV18, 39, 45, 59, 68, 70; clade 9: HPV16, 31, 33, 35, 52, 58, 67; ^*^clade 7 does not include HPV18 and clade 9 does not include HPV16 in these cross-tabulations.

In all, there were 710 individual HPV infections. Coinfection was equally common among LR-HPV genotypes (243 out of 268, 92%) and pHR-HPV genotypes (87 out of 101, 91%, *P*=0.21, *χ*^2^). Individuals infected with HR-HPV genotypes were somewhat less likely to be coinfected with a second HPV type (283 out of 341, 83%, *P*=0.006, *χ*^2^). The high-risk types that were most commonly seen in coinfection with HPV16 (clade 9) were HPV39 (28%), 45 (38%), and 59 (46%), belonging to HPV18 clade 7. Interestingly, HPV18 did not occur in either HPV39 or HPV45 (*P*⩽0.01, *χ*^2^), but was found in 13% of HPV59 infections (all clade 7). The high-risk types most commonly seen in coinfection with HPV18 were HPV56 (17%), 33 (14%), and 51 (14%), which did not differ from frequencies of HPV16 coinfection (*P*>0.7, *χ*^2^, for all). Figure 6 displays coinfection between HPV16 or HPV18 and the largest clades. HPV16-infected women were more often coinfected with clade 7 types (38%) than with similar clade 9 types (23%), although this did not reach statistical significance (*P*=0.06, *χ*^2^). Human papillomavirus type 18-infected women carried genotypes from clade 9 in 43% of cases, compared with similar clade 7 genotypes in 11% (*P*=0.02, *χ*^2^).

## Discussion

We have presented an overview of HPV genotypes encountered in unselected ASCUS and LSIL cases of all ages detected in a population-based cytology screening programme and then established the prevalence of multiple infections in these cases.

We found that HPV prevalence varied with age and there was a slight but non-significant increase in HPV prevalence in women over 45 years. Others have found similar age-related patterns and attributed this slight increase to menopausal hormonal and immunological changes that may facilitate HPV DNA detection or reactivate latent infections from exposures earlier in life (reviewed by Bosch *et al* (2008)). Such findings may also be related to sexual behaviour and thus to increased exposure, as many women in this age group have divorced and remarried (Stevenson and Wolfers, 2007). Our finding of an increasing proportion of low-risk types in post-menopausal women was also observed by Castle *et al* (2006). This observation, including the increase in multiple infections among women over 50 years, might also reflect changes in hormonal or immunological status occurring at menopause. Some evidence suggests a hormonal effect on oncogene expression, at least for HPV16 and 18, by action on progesterone/glucocorticoid-responsive elements in the long control region of the HPV genome (Cole and Danos, 1987; Gloss *et al*, 1989). However, more recent research has called such a mechanism into question (Ruutu *et al*, 2006), and would instead suggest hormonal effects impairing apoptosis of infected cells. Oral contraceptives and high parity are known risk factors for cervical cancer (Munoz *et al*, 2002). One common characteristic of these factors and menopause is a state of anovulation. To our knowledge, no studies have explored whether other ovulatory hormones such as gonadotropins, inhibin, or prostaglandins could influence HPV-induced carcinogenesis.

**Figure 7:**
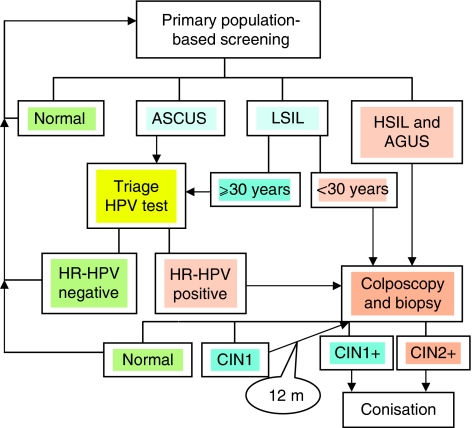
Suggested flowchart for triage HPV testing.

The average prevalence of HR-HPV was higher in LSIL (71%) than in ASCUS cases (49%), which is consistent with a previous meta-analysis (Arbyn *et al*, 2005). However, the LSIL cases had a significantly lower rate of HR-HPV than the rate reported by the ASCUS-LSIL-Triage-Study (ALTS) Group (82.9%) (ALTS, 2000). The ALTS group concluded that triage HPV testing of LSIL had limited potential for clinical decision making. The difference in HR-HPV prevalence might be due to different criteria for defining ASCUS and LSIL, and also to a difference in the mean age of the populations (24.9 years in ALTS and 33.6 years in this study). We used the LA assay covering 37 HPV types and defined 12 HPV types as high risk. ALTS used Hybrid Capture II to define the same 12 HR-HPV types and HPV68 as high risk. This approach should not introduce any major difference, as HPV68 without coinfection with another HR-HPV was found only in one case of ASCUS and in one case of LSIL in our material. Age-dependent HPV prevalence in women without cytological abnormalities is a known phenomenon (Castle *et al*, 2006; de Sanjose *et al*, 2007; Bosch *et al*, 2008). Age-dependent prevalence in LSIL has been observed and discussed by Ronco *et al* (2007), although they only compared women who were older or younger than 35 years. High-risk human papillomavirus prevalence was age-dependent in our LSIL cases, but more stable in ASCUS cases. When using 5-year intervals for age grouping, the only significant difference in HR-HPV prevalence between ASCUS and LSIL was found within the youngest (20–24 years) age group. In LSIL, the largest drop in HR-HPV prevalence was after the age of 30 years (83% in women <30 *vs* 60% in women ⩾30, *P*<0.001). These findings suggest that age 30, or even younger, is a suitable cutoff point for HR-HPV triage in LSIL, when consideration is given only to the prevalence argument. This is supported by a histological follow-up study of 112 of these cases by our group, in which 48% of women over 30 years with LSIL and 60% of women with ASCUS were HR-HPV negative and none of these had a histology result of CIN2+ (Froberg *et al*, 2008) and could therefore have avoided additional investigation. This implies that the number of follow-ups after low-grade or ambiguous cytology results could be cut in half, at least in women over 30 years of age. In Figure 7, we present a suggested flowchart for triage HPV testing.

Viral persistence is believed to be the most important risk factor for cervical cancer. Yet, this is true only for certain oncogenic types (Schiffman *et al*, 2005). Assessment of viral persistence requires HPV genotyping. Genotyping will also be necessary to monitor the effects of vaccination and to explore the importance of multiple infections. One of the most noteworthy findings in our study was the high frequency of multiple infections. Similar results were previously reported by Cuschieri *et al* (2004) in a general screening population from Edinburgh using a linear array assay capable of identifying 27 types and similar liquid-based sampling. We found that the prevalence of multiple infections was consistent with a quadratic function, with increasing prevalence among women over the age of 50 years. One may speculate whether this finding is caused by re-infection, triggering of a latent infection, or if detection is favoured by menopausal changes in the epithelium as mentioned above. Epidemiological cancer data from Statistics Sweden (SCB) reveal a second peak in cervical cancer incidence in the 65–69-year age group, which also suggests increased susceptibility in postmenopausal women. Multiple infection increases the probability of finding at least one carcinogenic type. It is still unclear whether coinfections with certain types can exert a synergistic effect on malignant transformation. Microdissection studies have suggested a polyclonal origin for squamous cervical cancer (Guo *et al*, 2001). The importance of multiple infections needs to be elucidated in longitudinal studies with histological confirmation of progressive CIN.

The observed elevated specific coinfection rates of HPV16 with certain other HPV types, such as HPV39, 45, and 59, may be because of differences in susceptibility to certain genotypes (all clade 7), as postulated by Liaw *et al* (2001). We found that HPV18-infected women were more likely to be coinfected with non-related clade 9 types than with similar clade 7 types, arguing against specific susceptibility. Rather, our data support cross-protection from infection with HPV from the same clade. Up to 60% of women with minor cytological abnormalities would potentially benefit from catch-up vaccination, provided full cross-protection can be assumed, as discussed by Castellsague *et al* (2008).

As far as we know, our study is the only one to describe the prevalence of HPV genotypes in ASCUS and LSIL cases among women of all ages in Sweden. The relative frequencies of individual HR-HPV types were similar to those found during primary screening in a Swedish multicentre study comprising 5696 women aged 32–38 years (Swedescreen) (Naucler *et al*, 2007). The Swedescreen group found that HPV16, 31, and 33 represented the highest population-attributable risk proportion in the development of CIN2+. Human papillomavirus type 18 was only the sixth most commonly identified HR-HPV type resulting in CIN2+. Worldwide, HPV33 is associated with the third highest odds ratio (OR 373.5) for squamous-cell cervical cancer, behind HPV16 (OR 434.5) and HPV59 (OR 419.5) (Munoz *et al*, 2003). Considering that odds ratios vary for different HPV types, genotyping is needed to estimate risk in the individual case as well as to plan clinical follow-up and vaccination strategy. We think our data support the statement of Cuschieri *et al* (2004) that ‘a broad spectrum test should be implemented until the true impact of the persistence of less common HR-HPV types in neoplastic progression is established’.

Minor cytological abnormalities associated with certain HPV genotypes may progress to cervical cancer. High-risk human papillomavirus prevalence is similar among women with ASCUS and LSIL after the age of 30 years, supporting an age limit of 30 years or even younger for triage testing in LSIL cases and no age limit in ASCUS cases, in order to improve the effectiveness of the screening programme. Human papillomavirus genotyping in longitudinal studies is needed to elucidate the importance of multiple infections for cancer progression, as well as cross-protection from vaccination.

## Figures and Tables

**Table 1:** Age-specific HR-HPV and multiple HR-HPV prevalence in the study population

	**Total number of women**		**HR-HPV**			**HR-HPV and HPV68**			**Multiple HR-HPV**		
	**Cytology**		**Cytology**			**Cytology**			**Cytology**		
**Age group (years)**	**ASCUS**	**LSIL**	**ASCUS No. (%)^a^**	**LSIL N (%)^a^**	***P* (*χ*^2^)**	**ASCUS No. (%)^a^**	**LSIL No. (%)^a^**	***P* (*χ*^2^)**	**ASCUS No. (%)^b^**	**LSIL No. (%)^b^**	***P* (*χ*^2^)**
20–24	18	55	8 (44)	47 (85)	<0.001	9 (50)	48 (87)	<0.001	5 (63)	23 (49)	0.74
25–29	23	50	15 (65)	40 (80)	0.17	15 (65)	40 (78)	0.17	7 (47)	18 (45)	0.91
30–34	17	35	10 (59)	22 (63)	0.78	10 (59)	22 (63)	0.78	4 (40)	9 (41)	0.73
35–39	16	36	10 (63)	23 (64)	0.92	10 (63)	23 (64)	0.92	2 (20)	8 (35)	0.66
40–44	21	18	6 (29)	10 (56)	0.09	6 (29)	10 (56)	0.09	0 (0)	3 (30)	0.41
45–49	13	15	7 (54)	7 (47)	0.70	7 (54)	7 (47)	0.70	2 (29)	1 (14)	1.0
50–60	11	13	3 (27)	8 (62)	0.20	3 (27)	8 (62)	0.20	2 (67)	1 (13)	0.30
All^c^	119	222	59 (49)	158 (71)	<0.001	60 (50)	159 (71)	<0.001	22 (38)	63 (40)	0.70

ASCUS=atypical squamous cells of undetermined significance; HR-HPV=high-risk human papillomavirus; LSIL=low-grade squamous intraepithelial lesion.

a Percentage of total number of women in the age group.

b Percentage of HR-HPV positive women in the age group.

c Two women are missing; one ASCUS case with HPV42 and one LSIL case with HPV52.

**Table 2:** Preventive potential of current HPV vaccines in Stockholm County, LSIL and ASCUS

	**Diagnostic group**			
	**All**	**ASCUS**	**LSIL**	
**HPV types**	**No. (%) (*n*=343)**	**No. (%) (*n*=120)**	**No. (%) (*n*=223)**	***P***
HPV+ (any type)	282 (82)	75 (63)	207 (93)	<0.001
HR-HPV+	217 (63)	59 (49)	158 (70)	<0.001
HPV16+	65 (19)	19 (16)	46 (21)	0.28
HPV18+	28 (8)	8 (7)	20 (9)	0.46
HPV16/18+	89 (26)	26 (22)	63 (28)	0.18
Clade 9+	154 (45)	44 (37)	110 (49)	0.02
Clade 7+	108 (31)	31 (26)	77 (35)	0.10
Clade 7/9+	206 (60)	58 (48)	148 (66)	0.001

ASCUS=atypical squamous cells of undetermined significance; HPV=human papillomavirus; LSIL=low-grade squamous intraepithelial lesion.

*P*-values indicate differences between ASCUS and LSIL cases.

Clade 7: HPV18, 39, 45, 59, 68, 70.

Clade 9: HPV16, 31, 33, 35, 52, 58, 67.
